# Ultrasonographically determined renal values and comparisons to serum biochemistry renal variables in aged semi-captive cheetahs (*Acinonyx jubatus*)

**DOI:** 10.1186/s12917-017-1234-x

**Published:** 2017-11-06

**Authors:** Robert M. Kirberger, Adrian S. W. Tordiffe

**Affiliations:** 1 Department of Companion Animal Clinical Studies, Faculty of Veterinary Science, University of Pretoria, Private Bag X04, Onderstepoort, 0110 South Africa; 2 Department of Paraclinical Sciences, Faculty of Veterinary Science, University of Pretoria, Private Bag X04, Onderstepoort, 0110 South Africa; 3 National Zoological Gardens of South Africa, Pretoria, South Africa

**Keywords:** Cheetah, Kidney function, Measurements, Diagnostic ultrasound

## Abstract

**Background:**

Cheetahs in captivity have a high prevalence of chronic renal diseases. We ultrasonographically evaluated the renal volumes, a variety of renal dimensions, interarcuate artery resistive indices (RI) as well as aortic diameters and the length of the ventral aspect of the 6th lumbar vertebrae in 27 aged semi-captive anesthetized cheetahs. Renal size, dimensions and ratios were compared to urine specific gravity, serum creatinine and urea values.

**Results:**

There were minimal differences for all values between left and right kidneys. Mean kidney length was 65.1 mm (range 55.2–76.9) with left kidney length ratios to L6 length being 1.60 (range 1.27–2.06) and to the aortic diameter 7.69 (range 4.54–10.72). Significant correlations between left renal length as well as length:L6 ratio to creatinine values were found ((r − 0.66) and (r − 0.60) respectively). The mean RI values of the different sedation/anesthetic protocols ranged from 0.46–0.55.

**Conclusions:**

Left renal length and L6 ventral vertebral body length as well as left kidney RI values should be routinely measured in all cheetah abdominal ultrasound examinations. These measurements, together with serum creatinine, urea and urine specific gravity values may be relatively sensitive indicators of early renal pathology in the absence of gross ultrasonographic changes.

## Background

The normal ultrasonographic anatomy of the cheetah liver, kidneys, urinary tract [1], adrenals [2] and female reproductive system have been described [3]. Diagnostic ultrasonography has been shown to be a valuable diagnostic tool, particularly under field conditions, to evaluate normal ultrasonographic abdominal anatomy as well as reproductive cyclical changes and has the potential to diagnose pathology as is routinely performed in domestic animals.

Chronic renal disease is known to be a common cause of morbidity and mortality in captive cheetahs. Nephropathies recorded at post-mortem include glomerulonephritis, glomerulosclerosis, renal fibrosis, acute tubular necrosis, interstitial nephritis, oxalate nephrosis, pyelonephritis and renal amyloidosis [4–10]. Of these, glomerulosclerosis and renal fibrosis tend to be the most common lesions, typically present in 70–80% of renal samples evaluated on histology in adult captive cheetahs [4, 5, 9].

Renal function can be evaluated by a variety of laboratory procedures including the determination of blood urea nitrogen (BUN) and creatinine concentrations. Urine protein to creatinine ratios as well as electrolyte fractional excretion ratios can also be determined. The serum concentration of symmetric dimethylarginine (SDMA) has also been shown to be a more useful indicator of early renal disease in domestic dogs and cats [11, 12], but its use has not yet been formally evaluated in cheetahs. Urine specific gravity and sediment evaluation are also potential indicators of renal disease. Some serum biochemical analytes have been reported specifically for cheetahs [13, 14]. A maximum serum creatinine concentration of 300 μmol/L has been suggested to reflect normal renal function in cheetahs [15] and this is supported by a more recent study in 66 apparently healthy cheetahs where a maximum concentration of 303 μmol/L was recorded [14].

A number of renal size imaging measurement techniques have been made in veterinary practice which could provide clinically useful information regarding possible underlying renal disease. These include computed tomography and radiography, which are usually not readily available under field conditions, and diagnostic ultrasound. The latter is readily accessible, has no ionizing radiation, does not require the use of intravenous iodine preparations to enhance renal visibility and can be performed repeatedly [16]. Ultrasound determined renal volumes in man have been shown to have a weak positive correlation with various glomerular filtration rate indices and could be a valuable prognosticator for the severity of chronic renal disease in resource poor human communities [17]. Similar findings could be applicable to cheetahs where sophisticated medical equipment is not always readily accessible. Additionally, serial volume determinations may be more accurate than single linear measurements to detect changes in renal size [18].

Radiographic renal size has been reported in normal captive cheetahs [19]. The ratio of the length of each kidney to the length of the second lumbar vertebral body (L2) was determined. There was no significant difference in this ratio between left and right kidneys with values ranging from 1.53–2.09. This was smaller than the ratio reported in ventrodorsal abdominal radiographs of domestic cats, which ranged from 1.9–2.6 times the length of L2 in neutered cats to 2.1–3.2 in entire cats [20].

Ultrasonographically determined renal size has been reported in dogs [21, 22], cats [23] as well as in the cheetah [1]. This may simplistically be done as renal length, width or height, or more accurately by determining renal volume. Volume measurements may be a more reliable indication of renal size than renal length. Numerous techniques have been described in dogs [18, 24] and cats [25]. Volumes can be determined using prolate ellipsoid geometric methods from linear and cross-sectional area measurements. Compared to the gold standard volume displacement method, the single plane area-length method had the best correlation (*r* = 0.94) [24]. Alternatively, and easier and faster to do, only linear measurements can be used to determine volume using a variety of similar formulas [18]. In the latter study the best correlation (*r* = 0.98) was obtained compared to the volume displacement method by using the following formula

$$ \mathrm{V}=\mathrm{L}\ \mathrm{X}\left(\frac{\mathrm{W}1+\mathrm{W}2}{2}\right)\mathrm{X}\left(\frac{\mathrm{H}1+\mathrm{H}2}{2}\right)\mathrm{X}\left(\frac{\pi }{6}\right) $$

where V = volume; L = maximum length W1 and H1 = width and height at the cranial pole and W2 and H2 the same measurements at the caudal pole.

More recently an ultrasonographically determined kidney-to-aorta ratio has been used to estimate renal size in normal dogs [26]. Renal length was compared to the longitudinal aortic maximum luminal diameter adjacent to the kidneys, just caudal to the origin of the left renal artery. Left and right renal lengths and ratios did not differ significantly with a mean ± standard deviation (SD) ratio of 7.3 ± 0.9 and range of 5.5–9.4.

No reference could be found to compare ultrasonographic renal length or volume to ultrasonographically determined lumbar vertebral body length. The value of diagnostic ultrasound in evaluating the vertebra and in the diagnosis of vertebral pathology, has been proven in dogs [27]. The ventral borders of the lumbar vertebrae can readily be identified, more so caudally, and the lumbosacral junction identified due to the angle of the sacral vertebral body to the lumbar vertebra in dogs and cats. Assuming seven lumbar vertebra in the cheetah, a caudal lumbar vertebra can be measured as a size comparison for renal length. The standard vertebra used for radiographic ratios, L2, may be more difficult to measure ultrasonographically due to the adjacent kidneys. The L7 length should not be used as in dogs it has been reported that it may be shorter than the remaining lumbar vertebra [20]. The L6 vertebra should thus be readily identifiable and measurable for comparison of renal length.

The Doppler resistive index (RI) is a non-invasive assessment of renal hemodynamics and has been advocated as a useful clinical parameter to detect real disease. The RI is calculated from the intrarenal arterial Doppler flow-velocity waveform as (peak systolic velocity – end diastolic peak velocity)/peak systolic velocity [28, 29]. Although initially believed to be a reflection of intrarenal disease, RI is a complex interaction between renal and systemic vascular wall properties and hemodynamics factors and in man it has been shown that there is a significant association between RI and central and peripheral pulse pressure [29].

Normal RI values for awake dogs are 0.63 ± 0.05 and 0.62 ± 0.05 for the left and right kidneys respectively [30]. In a study in dogs sedated with acepromazine maleate followed by diazepam and ketamine hydrochloride the values fell to 0.44 ± 0.06 and 0.45 ± 0.06 for left and right kidneys, a significant decrease compared to unsedated dogs [31]. In the latter study there was no significant difference between the left and right kidneys. Increased RI values in dogs have been attributed to total ureteral obstruction [30], acute renal disease [31] and Addison’s disease [32]. In 10 healthy domestic shorthaired cats sedated with acepromazine followed by intravenous ketamine hydrochloride (10 mg/kg) there was no significant difference between left and right arcuate artery RIs with a mean ± SD for the left kidney of 0.56 ± 0.06 and right kidney 0.59 ± 0.06 [33]. In another study of 50 healthy domestic shorthaired cats a similar anesthetic protocol was used but with the addition of xylazine (1 mg/kg) [23]. Their RI values were slightly lower at 0.52 ± 0.05 for the left kidney and 0.55 ± 0.05 for the right kidney. In another study comparing the renal RI in 8 awake versus isoflurane-anaesthetized cats, the awake cats had a RI of 0.55 ± 0.07 whilst the cats under anesthesia values were 0.93 ± 0.03 [34]. The duration of anesthesia prior to RI measurement was not stated. It was believed that the isoflurane produced increased intrarenal vascular resistance and decreased renal blood flow. In a study in awake Persian cats there was no statistical difference between left and right kidney interlobar RI values with a mean of 0.52 ± 0.06 [35]. In a more recent feline study comparing healthy cats and cats with renal disease, none of the cats were sedated or anaesthetized [36]. In the 24 healthy cats of various breeds the RI values differed significantly between left (0.59 ± 0.08) and right kidneys (0.54 ± 0.07) but implying no major difference between awake and anaesthetized cats when compared to the feline studies described earlier. However both kidneys showed significant increased RI value in cats with acute kidney injury (left 0.72 ± 0.08; right 0.74 ± 0.08) and chronic renal disease (left 0.73 ± 0.12; right 0.72 ± 0.11). One preliminary cheetah study measured RI values in 5 kidneys of 3 cheetahs, all on different anesthetic protocols, and they had a mean RI of 0.58 with a range of 0.47–0.68 [1].

The primary objective of this study was to determine a variety of renal variables in aged cheetahs by means of abdominal ultrasonography that could in future assist in detecting renal pathology induced dimension, volume or resistive index changes under field conditions. Additionally we sought correlations between ultrasonographically determined renal variables and serum creatinine and urea as well as urine SG to evaluate the possible benefit of these values.

## Methods

### Animals

The project was part of a long-term health and immune-competence study of captive cheetahs, Adult cheetahs, aged between 6 and 14 years, were received as orphaned wild cubs by the AfriCat Foundation near Otjiwarongo in Namibia. They were accommodated in fenced bushveld camps, ranging from 3.5 to 25 ha in size (average 11.42 ha) in small groups of two to six males and/or females that were either contracepted with deslorelin (Supralorin®, Virbac, Australia) implants or surgically sterilized to comply with Namibian legislative requirements. Their diet consisted of approximately 1.2–1.5 kg of donkey/horse meat and bones provided with a powdered multivitamin/mineral supplement (Predator supplement; V-Tech, Centurion, South Africa) on 6 days of the week and has been previously published [2, 3].

Twenty seven cheetahs were examined during a 5 day observation period in July 2016. Each cheetah was only examined once under general anesthesia. Their data (mean and range of age, weight and measurements) are presented in Table 1. The health examination carried out on each animal included a body condition assessment, dental and oral examination, gastric endoscopy, hematology and serum biochemistry, urinalysis and the abdominal ultrasound evaluation. All the animals included in the study displayed normal behavior prior to immobilization and were judged to be in good health. On gastric endoscopy, none of the cheetahs had macroscopic lesions typically associated with severe lymphoplasmacytic gastritis or any evidence of gastric ulcerations. Cheetahs were fasted for 48 h prior to anesthesia, but had free access to water. Cheetahs were immobilized with a dart gun using either a combination of tiletamine and zolazapam (Zoletil, Virbac, Halfway House, South Africa) (0.9–1.4 mg/kg) and medetomidine (Domitor, Zoetis, Sandton, South Africa) (0.03–0.045 mg/kg) or a combination of ketamine (Ketamine hydrochloride, Kyron Laboratories, Johannesburg, South Africa) (3.5–5.7 mg/kg) and medetomidine (0.03–0.45 mg/kg). For maintenance, one of two anesthetic protocols, either isoflurane in oxygen (end-tidal isoflurane concentration 1.1 ± 0.1%; Forane, Abbot Laboratories, Johannesburg, South Africa) or a propofol infusion (0.1 mg/kg/min; Fresenius Propoven 1%, Fresenius Kabi, Johannesburg, South Africa) with supplemental oxygen were used. Following the procedure, lasting on average between 60 and 90 min, the medetomidine was antagonised with atipamazole (Antisedan, Pfizer, Sandton, South Africa) (0.08–0.12 mg/kg).

**Table 1:** Cheetah age, weight, body measurement data (means and ranges)

	Males	Females	*P* value	Males and females
Number	17	10		27
Age (years)	8.9 (6–14)	9.9 (6–14)	0.47	9.3
Weight (kg)	40.4 (32.1–45.7)	35.0 (29.5–40.7)	0.003^*^	38.4
Body length (m)	0.99 (0.93–1.1)	0.94 (0.88–1.0)	0.004^*^	0.98
Shoulder height (m)	0.73 (0.68–0.77)	0.71 (0.68–0.74)	0.02^*^	0.72
Size index	0.73 (0.63–0.84)	0.67 (0.63–0.70)	0.001^*^	0.70

^*^Significant differences between males and females

### Body weights and measurements

All the cheetahs were weighed. Body length (BL) and shoulder height (SH) measurements were obtained with the animal in lateral recumbency using a 1.5 m flexible plastic measuring tape as published previously [2]. The BL measurement was taken from the occiput at the caudal edge of the skull to the base of the tail (at the fold created between the tail and the body when the tail is lifted to 90 degrees), ensuring that the measuring tape remained in contact with body along its entire length. The SH measurements were taken from the dorsal rim of the scapula to the middle of the metacarpal pad with the shoulder, elbow and carpal joints fully extended. A size index (SI) was calculated from using the formula shoulder height (m) X body length (m) similar to techniques described previously [37].

### Serum and urine variables

Blood samples were collected from the jugular vein within 20 min of immobilization using a 20 ml syringe and an 18 gauge needle. Blood was immediately transferred to a plain 6 ml serum tube (Vacutainer, Becton, Dickinson and Co, Sandton, South Africa) and allowed to clot on ice for 40 to 60 min. The tubes were centrifuged at 1500 rpm for 10 min after which the serum was pipetted off into 2 ml cryovials (Thermo Scientific, Germiston, South Africa) and analyzed immediately. Serum creatinine and urea concentrations were determined using the Vetscan® Kidney Profile Plus reagent rotor with an onsite Vetscan VS2® analyser (Abaxis Inc., Union City, USA).

Urine was collected within 20 min of immobilization by aseptic urethral catheterization using a 6 FG dog urinary catheter. Urine was aspirated into a 20 ml syringe and allowed to cool to room temperature before the SG was determined with a calibrated handheld refractometer (RHC-200ATC, Huake Instrument Co., Shenzhen, China).

### Ultrasonography

Each cheetah had its abdomen clipped before undergoing a dorsally recumbent trans-abdominal ultrasound examination of the abdomen. A portable ultrasound machine was used with a 3–6 MHz convex array transducer for the general abdominal examination and a 5–10 MHz linear array transducer set at 10 MHz for evaluation of the gastrointestinal tract and adrenal glands (Mindray Model M7 Vet, Shenzhen Mindray Biomedical from Lomaen, Johannesburg, South Africa) as published previously [2, 3].

**Fig. 1:**
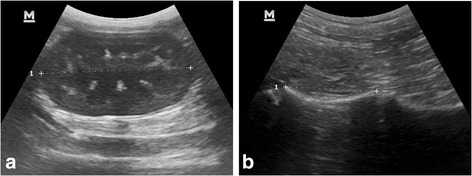
**a** Sagittal ultrasonographic image of a normal left kidney with cursors delineating its length. **b** Sagittal ultrasonographic image showing the ventral border of the caudal lumbar vertebra with cursors delineating the length of L6. On both side of the cursors are the intervertebral disc spaces and to the right of the cursors the ventral border of L7 can be seen. Ventral is at the top of the images and cranial is to the left

All measurements were made by one operator (RMK), recorded only once, except for RI, and were reported as mean, range and standard deviation. Each kidney had its length (Fig. 1a) and cranial and caudal pole maximum width and height measured. The length of the ventral aspect of ventral vertebral body of the second last lumbar vertebra (presumed to be L6), was measured (Fig. 1b). This was seen as a concave hyperechoic interface with acoustic shadowing with signal void in the adjacent disc spaces. The aortic luminal diameter was measured just caudal to the left renal artery exit from the longitudinal plane as described previously by Mareschal et al. [26]. The renal volumes were then calculated by means of the prolate ellipsoid technique using the average of the cranial and caudal pole measurements as described by Nyland et al. [18]. Ratios of the renal length and volume to the length of L6, width of the aorta and SI were then determined.

The RI was determined by placing the pulsed wave sampling gate of the 10 MHz linear array transducer in an interlobar artery at the corticomedullary junction region using color flow Doppler. The lowest peak velocity scale was selected that allowed the wave form to be traced without aliasing. Three similar wave forms were measured and the mean RI calculated. As numerous clinical procedures had to be performed on the cheetahs only left renal values were determined to limit anesthetic time. The RI measurements were compared between the two immobilization darting protocols and the two anesthesia maintenance protocols.

Since male cheetahs were significantly larger than females, the renal measurements were also analyzed using values that were corrected for body size, by dividing the various renal values by the SI.

### Statistical analyses

Data were reported as means ± SDs unless otherwise indicated. All measurements, other than age, serum creatinine and urea were normally distributed as assessed by Shapiro-Wilk’s test (*P* > 0.05). Homogeneity of variance was demonstrated in males and females for all measurements, except for SI, using Levene’s test for equality of variance. Unpaired t-tests were used to compare the weights, body size, and renal measurements of males and females. Paired-sample t-tests were used to evaluate the differences between left and right renal measurements. A Mann-Whitney U test was run to compare the ages, serum creatinine and urea values of males and females. Spearman’s rank correlations were used to evaluate the influence of age, serum urea, serum creatinine and urine SG on the various renal measurements. All analyses were performed with SPSS version 23.0 for Windows (IBM Corporation). Statistical significance was two-tailed and defined as *P* < 0.05 in all cases.

## Results

Cheetah weights, body measurements and size index differed significantly between males and females (Table 1). Serum and urine variables did not differ significantly between males and females and there were no significant correlations to cheetah age (Table 2).

**Table 2:** Cheetah renal function variables (means and ranges) using Vetscan VS2

	Males	Females	Males and females^a^
Number	17	10	27
Serum creatinine (μmol/L)	240 (108–463)	248 (179–330)	243 (108–463)
Serum urea (mmol/L)	12.7 (7.3–22.7)	12.8 (7.9–18.3)	12.73 (7.3–22.7)
Urine specific gravity	1.052 (1.025–1.072)	1.054 (1.042–1.062)	1052 (1025–1072)

^a^There were no significant differences between males and females and data were thus combined

General abdominal ultrasonography only detected incidental splenic myelolipomas in 24 cheetahs ranging in number from 1 to more than 50 in an individual cheetah and these varied in diameter from 1 to 14.9 mm.

The kidneys appeared ultrasonographically normal and the measurements could readily be made as could the aortic luminal diameters and length of L6 (Tables 3, 4). Significant differences between male and female ultrasound measurements could only be found for aortic diameter (*P* = 0.006), right renal volume (*P* = 0.02) and right renal volume:L6 length (*P* = 0.03) with values significantly larger in males compared to females. Males had significantly longer kidneys than females but when corrected for weight this was not significant.

**Table 3:** Cheetah renal measurements in mm or mm^3^

	Measurement (mm)	*N*	Mean	SD	Minimum	Maximum
Left kidney	Length	27	65.1	5.5	55.2	76.9
	Cranial pole width	26	42.1	4.4	30.1	52.0
	Cranial pole height	26	38.5	3.8	32.8	47.4
	Caudal pole width	27	41.4	4.4	32.6	49.0
	Caudal pole height	27	36.6^a^	4.7	28.3	46.9
	Volume	24	52.4	10.5	35.8	72.67
Right kidney	Length	26	65.8	4.9	54.9	78.3
	Cranial pole width	26	42.8	4.1	35.1	51.3
	Cranial pole height	26	40.3	3.3	33.3	48.2
	Caudal pole width	26	41.6	4.1	35.2	50.9
	Caudal pole height	26	39.1^a^	3.1	33.9	46.8
	Volume	24	57.0	9.2	39.1	78.9
Both kidneys^b^	Length	53	65.4	5.2	54.9	78.3
	Cranial pole width	52	42.5	4.2	30.1	52.0
	Cranial pole height	52	39.4	3.6	32.8	48.2
	Caudal pole width	53	41.5	4.2	32.6	50.9

^a^Significant difference between left and right renal measurements

^b^Values combined for left and right kidneys as they were not significantly different

**Table 4:** Cheetah aorta, L6 and size index (SI) values and associated ratios

	Measurement or ratio	*N*	Mean	SD	Minimum	Maximum
	Aorta diameter (mm)	27	8.6	1.3	6.5	12.3
	L6 length	27	40.6	3.1	33.9	48.8
Left kidney	Length:Ao	25	7.69	1.21	4.54	10.72
	Volume:Ao	24	6.25	1.39	2.91	8.55
	Length:L6	25	1.60	0.19	1.27	2.06
	Volume:L6	24	1.31	0.29	0.87	1.91
	Length:SI	27	93.2	9.8	71.75	108.8
Right kidney	Length:Ao	24	7.82	1.18	4.59	10.23
	Volume:Ao	24	6.78	1.17	4.27	10.38
	Length:L6	24	1.62	0.17	1.29	1.96
	Volume:L6	24	1.42	0.26	0.94	1.94
	Length:SI	27	94.0	8.7	72.65	107.6
Both kidneys^a^	Length:Ao	49	7.75	1.62	4.54	10.72
	Volume:Ao	48	6.51	1.29	2.91	10.38
	Length:L6	49	1.61	0.18	1.27	1.96
	Volume:L6	48	1.36	0.28	0.87	1.94
	Length:SI	54	93.6	9.1	71.75	108.8

^a^Values combined for left and right kidneys as they were not significantly different

In comparing left and right kidney dimensions only the caudal pole height differed significantly between the two kidneys (Table 3). In calculating ultrasonographically determined ratios there were no significant differences between the two kidneys (Table 4). Interestingly, dimensions and ratios of the right kidney values were always slightly larger than those of the left kidney.

A limited number of RI values could be measured (Table 5) as anesthetic protocols had to be adapted due to several cheetahs developing post darting hyperthermia and the long recovery times of the propofol infusion anesthetic maintenance protocol. Of the 15 kidneys evaluated 3 had an average of 2 rather than 3 measurements. The ketamine/medetomidine maintained with propofol was the only regimen with a mean value under 0.50 whilst the other 3 protocols ranged from 0.51 to 0.55.

**Table 5:** Cheetah RI values using different immobilization and anesthetic maintenance protocols

Immobilization	Maintenance	*N*	Mean	SD	Range
Ketamine/Med^a^	Propofol	4	0.46	0.01	0.44–0.47
Ketamine/Med	Isoflurane	1	0.51	–	–
Zoletil/Med	Propofol	6	0.55	0.07	0.46–0.66
Zoletil/Med	Isoflurane	4	0.54	0.07	0.45–0.61
All combined	All combined	15	0.52	0.07	0.44–0.66

^a^medetomidine

**Fig. 2:**
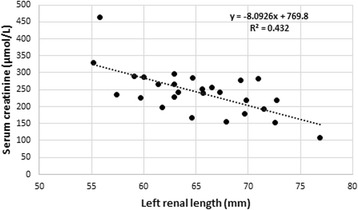
Scatterplot of left renal length and serum creatinine concentrations

**Fig. 3:**
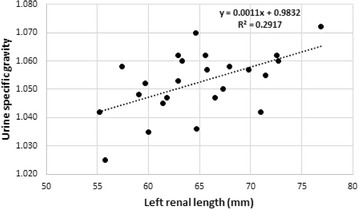
Scatterplot of left renal length and urine specific gravity values

**Fig. 4:**
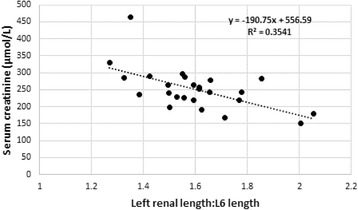
Scatterplot of left renal length:L6 and serum creatinine concentrations

Significant correlations between renal measurements and serum creatinine and urea concentrations, as well as urine specific gravity values were found (Table 6). In particular left renal length correlated well with creatinine (*r* = −0.66, Fig. 2) and urine specific gravity (*r* = 0.54 Fig. 3) whereas left renal length:L6 correlated well with creatinine (r = −0.6) and urea (*r* = −0.62, Fig. 4). Left renal length:SI correlated well with creatinine (*r* = −0.63) and urine specific gravity (*r* = 0.56). None of the renal or other measurements correlated significantly with age.

**Table 6:** Significant Pearson’s correlations between renal measurements/ratios and serum creatinine, serum urea and urine specific gravity

	Renal measurement	Serum creatinine *N = 27*		Serum urea *N = 27*		Urine SG *N = 24*	
		*r*	*P* value	*r*	*P* value	*r*	*P* value
Left kidney	Length	−0.66	< 0.0005	−0.39	0.043	0.54	0.006
	Length:Size Index	−0.66	< 0.0005	−0.52	0.006	0.60	0.002
	Length:Ao d	−0.65	< 0.0005	−0.54	0.006	0.50	0.017
	Volume:Ao d	−0.51	0.011	−0.62	0.001	0.39	NS
	Volume:L6	−0.42	0.043	−0.58	0.003	0.35	NS
	Length:L6	−0.60	0.002	−0.62	0.001	0.53	0.012
	Cranial height	−0.40	0.044	−0.25	NS	0.43	0.043
	Volume	−0.35	NS	−0.55	0.006	0.38	NS
Right kidney	Length	−0.44	0.023	−0.31	NS	0.38	NS
	Length:Size Index	−0.53	0.006	−0.47	0.015	0.46	0.025
	Length:Ao d	−0.52	0.009	−0.48	0.017	0.40	NS
	Length:L6	−0.46	0.025	−0.56	0.004	0.43	0.045
	Volume:Ao d	−0.36	NS	−0.43	0.034	0.16	NS

*NS* Not significant

## Discussion

This was a cohort of 27 aged semi-captive cheetahs with a mean age of 9.3 years (combined male and female). There were significant differences between size and weight measurements between males and females as reported previously [38, 39]. The serum and urine variables, as well as most of the ultrasonographic measurements, did not differ significantly between males and females and there were no correlations between any of these variables and the ages of the animals. Ultrasonographically determined lengths, widths and volumes, as well as renal length:L6 and renal length:Ao did not differ significantly between left and right kidneys but the caudal heights were significantly different (*P* = 0.02).

The mean serum creatinine concentrations for male and female cheetahs in our study (240 and 248 μmol/L respectively), were slightly higher than the mean values reported previously by our group in captive (184 μmol/L, mean age = 8.5 years) and free-ranging cheetahs (169 μmol/L, mean age = 4.0 years) [14], but similar to the mean concentration (240 μmol/L) in thirteen captive cheetahs with a mean age of 5.02 years, as reported by Holder et al. [13]. Two of the cheetahs in our study had serum creatinine concentrations above the upper limit of 300 μmol/L suggested by Lane et al. [15]. Interestingly these two individuals had the lowest left renal length measurements (Fig. 1), but neither showed any signs of clinical renal failure (weight loss, polyuria/polydipsia or loss of appetite). Our slightly higher creatinine values could be ascribed to the relatively old age of our cohort of cheetahs. However, the serum variables for the comparative studies were determined by different analytical methods which could also have resulted in different reference intervals. The serum urea and urine specific gravity values in our study were comparable with those previously reported [13, 14].

Our renal size variables compared well to a previous ultrasonographic study of 13 younger (mean age 3.9 years) captive bred cheetahs where mean length was 63.9 (range 52 - 74 mm) compared to our slightly longer values of 65.4 (54.9–78.3) [1]. The other size variables were also similar but were measured at different locations and could not be directly compared. Ultrasonographically determined renal length can thus be accurately measured and the technique to compare left renal length to the ultrasonographically determined L6 length would appear to be a useful dimension to detect smaller kidneys potentially associated with subclinical diffuse renal disease. The kidney length:L6 had one of the lowest relative standard deviation values of all the ratios emphasizing the value of utilizing the kidney length:L6 length as an intra cheetah evaluation of renal size irrespective of its weight.

Our study found interesting correlations between several renal measurements in relation to creatinine, urea and urine specific gravity (Table 6). Left renal length and the left renal length:SI ratios correlated most strongly with serum creatinine values (*r* = 0.66, *P* < 0.0005 in both cases), followed by the left renal length:aortic diameter (*r* = 0.65, *P* = < 0.0005) and left renal length:L6 ratios (*r* = 0.60, *P* = 0.002). Shorter left renal length and a lower ratios thus related to increasing creatinine and urea levels and decreasing specific gravity values. Given the high incidence of renal pathology in captive cheetahs, these ratios may well be indicative of subclinical chronic renal disease and may be useful measurements to make over time to asses renal changes in cheetahs. The results also suggest that reference intervals established for serum creatinine, urea and urine specific gravity in captive cheetahs are likely to include animals with subclinical renal disease and that serum creatinine values are perhaps more sensitive to subtle changes in renal mass than previously thought.

Furthermore, although the common renal diseases of the cheetah, glomerulosclerosis, amyloidosis and fibrosis, may be ultrasonographically detected in the advanced stage as increased cortical echogenicity as seen in dogs and cats, hyperechoic cortices may have many causes. A recent ultrasonographic – histologic study in dogs and cats determined that mean gray value (= echogenicity) was a poor test to distinguish normal from pathological kidneys with a sensitivity of 58.3% and specificity of 59.8% in dogs but with slightly better values in the domestic cat of 80.6% and 56% respectively [40]. Based on our ultrasonographic study, where no cheetahs had evidence of increased cortical echogenicity, renal size could potentially be a more important indicator of early renal pathology.

The RI values for the various darting and anesthesia maintenance protocols obtained in 15 cheetahs ranged from 0.44 to 0.66 with a mean overall value of 0.52. These values are comparable to those of domestic cats under different anesthetic protocols which mean values ranged from 0.52–0.90 (33, 34]. These values did not differ significantly from values in awake cats [36]. Significant increased RI values have been shown in domestic cats with acute renal injury and chronic renal disease with mean values between 0.72 and 0.74 [36]. It is likely that these elevated RI values will also elevate to values >0.65 in some cheetah renal diseases and this could be a useful clinical field parameter to detect early renal pathology. However additional clinical data will be required to support this. Our field work will now routinely determine the RI values in at least the left kidney.

Overall left renal values appeared to have lower standard deviations than the right kidney thus making it the kidney of choice to measure ultrasonographically when evaluating kidneys for chronic renal disease likely to be affecting both kidneys. The right kidney is located more cranially and partially subcostally making ultrasonographic accessibility difficult when compared to the left kidney, which is easy to find, as the spleen may act as an acoustic window. We have reported similar findings when evaluating cheetah adrenals ultrasonographically, where the left adrenal was also easier to find and measure than the right, thus resulting in more accurate adrenal measurements with lower standard deviations [2].

## Conclusions

Left renal length and L6 ventral vertebral body length as well as left kidney RI values should be routinely measured in all cheetah abdominal ultrasound examinations. These values, together with serum creatinine, urea and urine specific gravity values may be relatively sensitive indicators of early renal pathology in the absence of gross ultrasonographic changes.

## Abbreviations

Ao: Aorta
BL: Body length
L: Lumbar vertebra
RI: Resistive index
SD: Standard deviation
SG: Specific gravity
SH: Shoulder height
SI: Size index
